# High-Resolution Transcranial Electrical Simulation for Living Mice Based on Magneto-Acoustic Effect

**DOI:** 10.3389/fnins.2019.01342

**Published:** 2019-12-13

**Authors:** Xiaoqing Zhou, Shikun Liu, Yuexiang Wang, Tao Yin, Zhuo Yang, Zhipeng Liu

**Affiliations:** ^1^Chinese Academy of Medical Sciences & Peking Union Medical College, Institute of Biomedical Engineering, Tianjin, China; ^2^College of Medicine, State Key Laboratory of Medicinal Chemical Biology, Nankai University, Tianjin, China

**Keywords:** transcranial focused electrical simulation, transcranial magneto-acoustic stimulation, animal experiment, transcranial ultrasound stimulation, neuromodulation

## Abstract

Transcranial electrical stimulation is an important neuromodulation tool, which has been widely applied in the cognitive sciences and in the treatment of neurological and psychiatric diseases. In this work, a novel non-invasive method of transcranial electrical stimulation with high-resolution transcranial magneto-acoustic stimulation (TMAS) method has been tested experimentally in living mice for the first time. It can achieve spatial resolution of 2 mm in the cortex and even in the deep brain regions. The induced electrical field of TMAS was simulated and measured using a test sample. Then, an animal experimental system was built, and the healthy as well as Parkinson’s disease (PD) mice were simulated by TMAS *in vivo*. To investigate the effect of transcranial ultrasound stimulation (TUS) at the same time as TMAS, a TUS group was added in the experiments and its results compared with those of the TMAS group. The results not only demonstrate the high-resolution ability and safety of TMAS, but also show that both TMAS and TUS improved the synaptic plasticity of the PD mice and might improve the spatial learning and memory ability of the healthy mice and the PD mice, although the improvement performance of the TMAS group was superior to that of the TUS-group. Based on the *in vivo* TMAS studies, we propose that TMAS functions as a dual-mode stimulation combining the electric field of the magneto-acoustic effect and the mechanical force of TUS. Our results also provide an explanation of the mechanism of TMAS. This research suggests that future use of US stimulation in magnetic resonance imaging (MRI)-guided studies should involve careful consideration of the induced magneto-acoustic electrical field caused by the static magnetic field of MRI.

## Introduction

Transcranial electromagnetic stimulations have been widely applied in the cognitive sciences and in the treatment of neurological and psychiatric diseases (Fregni and Pascual-Leone, 2007; Bergmann et al., 2016; Grossman et al., 2017). They directly create electrical fields (E-fields), which influence the electrical activities of neurons in the brain by electrical current injection or magnetic induction. At present, transcranial magnetic stimulation (TMS) (Rotenberg et al., 2014) is the most popular form of non-invasive transcranial electromagnetic stimulation. It utilizes magnetic energy that passes through the skull without attenuation to modulate neural activities and has been used to treat various brain disorders, including tremors, depression, seizures, schizophrenia, pain, and tinnitus (Theodore et al., 2002; Loo and Mitchell, 2005; Borckardt et al., 2006; Lu et al., 2015; Amiaz et al., 2016). However, given that electromagnetic fields obey Laplace’s equation, it is impossible to create local maxima in the field intensity, no matter what the configuration of the source coils (Norton, 2003). TMS often does not achieve adequate spatial resolution on the millimeter scale, and it is helpless for specific activation of neuronal cells in a region less than 5 mm (Deng et al., 2013; Markovitz et al., 2015; Guo et al., 2018). In addition, because its magnetic focusing becomes poorer as the penetration depth increases, TMS is not suitable for the stimulation of deep brain tissues. Usually, deep brain stimulation (DBS) (Pereira et al., 2007; Khabarova et al., 2018) is used to stimulate deep brain areas accurately by placing electrodes in the inner area of the brain. However, this involves neurosurgical surgery, which makes it a highly invasive method.

Norton (2003) proposed the novel idea of using ultrasound (US) for focused electrical stimulation by Lorentz forces. This was expected to achieve a focused electrical field with spatial resolution of millimeter scale in the cortex and even in the deep regions. According to Norton, the stimulating electrical field would be induced not by an electric or magnetic field directly, but by the combined action of a US wave and a static magnetic field based on the magneto-acoustic effect. The ion motion created by an ultrasonic wave would form a Hall electric field generated by Lorentz forces. In theory, the induced electric field is consistent with the focused ultrasonic field in a homogeneous medium. In 2006, Zhang et al. (Hongmiao et al., 2005) tested Norton’s theoretical format. They analyzed the electrical signals in a gel saline phantom resulting from a combination of ultrasonic signals and static magnetic field and found that the electrical signals were consistent with the ultrasonic signals in the frequency domain. Yuan et al. (2016) focused on the neuronal firing pattern of Norton’s method, which they called transcranial magneto-acoustic stimulation (TMAS). They investigated the stimulatory mechanism of TMAS using the Hodgkin–Huxley neuron model and presented simulation results for the neuronal firing pattern. Zhang et al. (2018) improved the stimulatory mechanism of this method by considering the membrane capacitance of neuron changes under an ultrasonic radiation force, based on the Izhikevich model, and also produced simulation results for the firing activity of neurons.

In this paper, we evaluate this novel high-resolution transcranial electrical simulation method, which we also call TMAS, using simulations and measurements of the induced electrical field, as well as *in vivo* animal experiments for the first time. Here, a TMAS system was built and the intensity and distribution of the focused electrical field were measured using a short copper wire. Based on this system, we designed parameters to form a proper stimulated electrical field to stimulate both healthy mice and mouse models of Parkinson’s disease (PD) by TMAS *in vivo*. Behavioral tests and electrophysiology studies were performed to explore the biological effects of this novel TMAS.

Moreover, we achieved high-resolution electrical stimulation by utilizing a highly collimated ultrasonic beam in the energy of a static magnetic field, showing that the TMAS process inevitably contains US stimulation, owing to their similar systems and physical principles. In recent years, multiple studies have demonstrated that US can successfully modulate neural activity in the brain at different frequencies (0.3–5 MHz) and different intensity levels (0.02–1000 W/cm^2^) in wild-type animals and humans (Tufail et al., 2011; Baek et al., 2017; Sato et al., 2018). Here, we also investigated the effects of transcranial ultrasound stimulation (TUS) on the TMAS process. In the animal experiments, a TUS treatment group was compared with the TMAS treatment group with respect to the behavioral and electrophysiology results. The electrophysiology results show that both TMAS and TUS improved the synaptic plasticity of the PD mice. And the behavioral results suggested that both TMAS and TUS might improve the learning and memory ability of both the healthy mice and the PD mice as supplementary, due to the limitation by the sample size of the behavioral tests (3 or 4 mice per treatment group). In addition, both the healthy and the PD mice, the TMAS group showed better performance than the TUS group. Based on the *in vivo* results, we suggest that TMAS is a complex stimulation combining the electric field of the magneto-acoustic effect and the mechanical force of TUS. The results also provide an explanation of the mechanism of TMAS. Finally, we suggest that the use of TUS in magnetic resonance imaging (MRI) scanners may in future require consideration of the induced magneto-acoustic electrical field (E-field) caused by the strong static magnetic field of MRI.

## Materials and Methods

### Theory of TMAS

**FIGURE 1:**
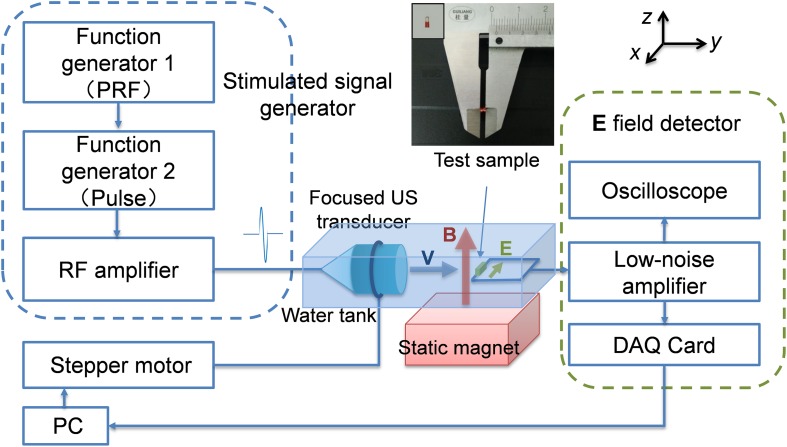
E-field distribution detection system for TMAS.

The TMAS method is based on the magneto-acoustic effect of conductive tissue, illustrated as the central region in Figure 1. The longitudinal particle motion of an ultrasonic wave causes the ion to oscillate back and forth in the medium with velocity **V**. In the presence of a static magnetic field, **B**_0_, the ions are subjected to the Lorentz force **F**_L_ and form the equivalent electrical field **E**, i.e., the simulated electrical field of TMAS:

(1) $$\left\{ \begin{array}{c} F_{\text{L}\,z}=q\,V_{y}\,B_{0\,x}\\ F_{\text{E}\,z}=q\,E_{z} \end{array}\right.$$

Considering the F_E*z*_ = F_L*z*_:

(2) $$E_{z}=V_{y}\,B_{0\,x}$$

Eq. 2 can also be written as the vector expression: **E** = **V**×**B**_0_.

As **J** = σ**E**, where σ is the conductivity of the tissue, the current density in the tissue can be written as:

(3) $$J_{z}=\sigma \,V_{y}\,B_{0\,x}$$

For the plane cosine wave, the relationship between the acoustic pressure *P*_*y*_ and the vibration velocity *V*_*y*_ can be expressed as:

(4) $$P_{y}=\rho \,c_{s}\,V_{y}$$

where ρ is the density of the tissue and *c*_*s*_ denotes the acoustic speed in a tissue. Then Eq. 1 can be written as:

(5) $$\left\{ \begin{array}{c} E_{z}=\frac{1}{\rho \,c_{s}}\,P_{y}\,B_{0\,x}\\ J_{z}=\frac{\sigma }{\rho \,c_{s}}\,P_{y}\,B_{0\,x} \end{array}\right.$$

Using Eq. 5, it can be shown that the induced (or coupled) electrical field **E** is simultaneously perpendicular to the direction of the static magnetic field **B**_0_, as well as the acoustic field propagation. The intensity of **E** is related to the values ρ, σ, *c*_*s*_ of the tissue, *B*_0_, and the applied acoustic pressure *P*. The distribution of **E** is consistent with the distribution of an acoustic field in a homogeneous medium. This means that we can obtain high-resolution electrical stimulation by utilizing a highly collimated ultrasonic beam and the energy of the static magnetic field. In addition, the stimulation of cortical tissue can be highly localized in this way, as well as being achieved at greater depths in the brain.

### Experimental Setup for TMAS

To measure and evaluate the distribution and intensity of the E-field in TMAS, an experimental system was set up, as shown in Figure 1.

Function generator 1 (TFG6920A, Shuying, China) was used to trigger US pulses and to define the pulse repetition frequency (PRF) and the pulse number of the stimulus waveform. Function generator 2 (AFG3252, Tektronix, United States) was used to define the US frequency and the number of cycles per pulse. The pulsed signals were fed to an RF amplifier (GA2500, RITEC, United States) and used to stimulate a focused US transducer (FP-1M, IOA-AC, China) with a central frequency of 1 MHz and a bandwidth of 400 kHz. The focus size and focal length of the transducer were 2 and 23 mm, respectively. The US transducer was positioned by a holder linked to a three-dimensional stepper motor. The static magnetic field was provided by a permanent magnet. The magnetic field strength *B*_0_ of the magnet was detected by a Gauss meter (Model 475, Lakeshore, United States). The test sample was a 2 mm copper wire located in an adjusting seat. The induced electric signals of the test sample were amplified by a low-noise amplifier (5660C, Olympus, Japan) and acquired using an oscilloscope (MSO4104, Tektronix, United States) or a data acquisition card (PXI-5122, NI, Japan). The sample and the transducer were both immersed in deionized water as an acoustic coupling agent.

**FIGURE 2:**
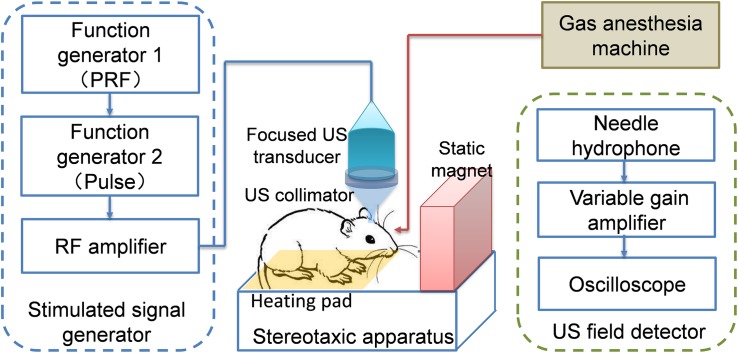
Schematic diagram of the TMAS system for small animals.

To evaluate the TMAS method *in vivo*, an experimental system for small animals based on that in Figure 1 was set up, as shown in Figure 2.

The stimulated signal generator part was the same as Figure 1. In this case, the US transducer was positioned using a stereotaxic instrument (SR-6M, Narishige, Japan) and used to perform brain stimulation of targets in the mouse brain. To acquire better localization, a US collimator filled with US coupling gel was used between the US transducer (FP-1M, IOA-AC, China) and the mouse’s head. The mouse was laid on a heating pad and fixed by a mouse holder. A permanent magnet was set up beside the mouse. The direction of the magnetic field was perpendicular to the US direction; this setup could produce an ionic current (i.e., a stimulated E-field) pulse along the sagittal direction in the mouse brain. A gas anesthesia machine (R580S, RWD, China) was used to provide anesthesia for the mouse during the TMAS treatments. To acquire the US intensity in real time, a US field detector containing a standard needle hydrophone (NH-1, IOA-AC, China) and a data acquisition device was established.

For TMAS, the static magnetic field is an indispensable element. Without the static magnetic field, the TMAS system in Figure 2 would be a normal TUS system. Therefore, we could set up or remove the permanent magnet to perform TMAS and TUS, respectively.

### Simulation of the Induced Electrical Field in TMAS

**FIGURE 3:**
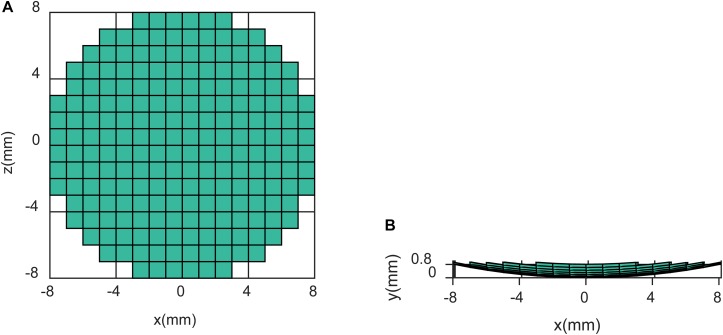
Specification of the transducer at **(A)** the *x*–*z* section and **(B)** the *x*–*y* section.

To prove the relationship between the E-field and the US field, we simulated the US field distribution of the transducer used in our experiments based on the wave equation written as Eq. 6 and the induced E-field distribution from Eq. 5.

(6) $$\nabla^{2}p\,\left(r,t\right)-\frac{1}{c_{s}^{2}}\,\frac{\partial^{2}p\,\left(r,t\right)}{\partial t^{2}}=S\,\left(r,t\right)$$

where *p*(**r**,*t*) denotes the acoustic pressure at location **r** and time *t*, *c*_*s*_ is the acoustic speed in a medium and *S*(**r**,*t*) denotes the source item induced by the US transducer. The intensity of the E-field at different US pressures was also simulated. The specification of the transducer used in the simulation is shown in Figure 3.

In the simulation, a homogeneous medium with the same acoustic and electrical parameters as biological tissue was employed. Values of ρ = 1000 kg/m^3^, *c*_*s*_ = 1450 m/s, and σ = 1S were used in Eq. 5.

### Measurement of the Induced Electrical Field in TMAS

To validate the simulation results, the distribution and intensity of the E-field in TMAS was measured using the system shown in Figure 2. The US pressure field distribution of the transducer and the induced E-field distribution were measured and compared. The distribution of the US pressure field was measured using an Acertara acoustic measurement system (AMT, Acertara, United States). In this system, the ultrasonic transducer is positioned by a holder linked to a three-dimensional stepper motor and a hydrophone is used to measure the ultrasonic pressure signal in 3D space at the specified steps. The distribution of the induced E-field was measured with 2 mm steps at the *x*–*y* section and *x*–*z* section. We also measured the induced voltage of the test sample at different US pressures by changing the stimulated signal.

### TMAS Treatment for Living Small Animals

Adult specific-pathogen free (SPF) male C57BL mice of 8 weeks of age were purchased from the Experimental Animal Center of Chinese Academy of Medical Sciences. All experiments were performed according to protocols approved by the Committee for Animal Care of Nankai University and in accordance with the practices outlined in the National Institutes of Health Guide for the Care and Use of Laboratory Animals.

#### Healthy Mice Stimulation

First, nine healthy mice were stimulated using the system shown in Figure 2. In order to investigate the relative contribution of TUS to the TMAS effect, the nine mice were divided into three groups in random: TMAS, TUS, and control (Con).

The mice (20–22 g) were anesthetized with 4% isoflurane, then fixed by the mouse holder of the stereotaxic apparatus. US coupling gel was applied and gently wiped on the mouse’s scalp. During the stimulation, the mice were subjected to light anesthesia with 1% isoflurane. The TMAS group received TMAS treatment with US stimulation focused at the substantia nigra of the mouse brain [centered at anteroposterior (AP) −3.4 mm, mediolateral (ML) 1.5 mm, dorsoventral (DV) 4.5 mm] within a static magnetic field of 0.2 T, while the TUS group received TUS treatment at the same target region without the static magnetic field. The con groups received sham stimulation by turning off the US stimulated signal on the mouse head, with the same static magnetic field and the same conditions as the TMAS and TUS groups.

**FIGURE 4:**
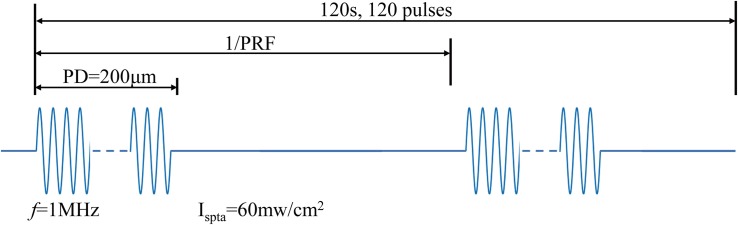
Ultrasound stimulated signal for TMAS in live animal experiments.

Considering the induced electric field intensity and the security of ultrasonic treatment, the US stimulated signal was chosen as in Figure 4.

The frequency of the US stimulated signal was 1 MHz. The pulse duration (PD) was 200 μm with 1 Hz PRF. We measured the intracranial US pressure of a mouse using the US field detector shown in Figure 2. The intracranial US pressure *P*_in_ was approximately 3 MPa, and the spatial-peak temporal-average intensity I_spta_ was approximately 60 mW/cm^2^. The duration of stimulation was set to 2 min and contained 120 pulses. All the mice received TMAS, TUS, or sham stimulation once each day for consecutive 10 days. After 10 days of treatment, animal behavior tests were carried out, including the elevated plus-maze test (Okonogi et al., 2018), the open field behavior test (Aulich, 1976) and the Morris Water Maze (MWM) test (Vorhees and Williams, 2006) to assess the emotion and the learning and memory abilities of the healthy mice.

#### Behavioral Test Methods

The elevated plus-maze was made of black plastic boards and consisted of four arms (two open arms without walls and two enclosed by 15-cm-high walls) 30 cm long and 5 cm wide. Each test began with a mouse placed on the central platform and made to face an open arm. Mouse activities were recorded with a camera located 100 cm above the maze and analyzed on a computer.

The open field arena was made of black plastic (30 cm × 30 cm × 15 cm). Each test began by placing a mouse at a central point, and mouse activities were recorded for 5 min with a video camera located 100 cm above the arena. The arena was equally divided into 16 squares on the computer, and the four squares in the center were defined as the “center” area.

The MWM system (RB-100A type; Beijing, China) contained a 90-cm-diameter swimming arena filled with warm water (25°C ± 1°C) that was stained white with non-toxic TiO_2_ powder. The swimming activities of mice were recorded by a video camera and analyzed by a computer. On the computer software, the swimming arena was equally divided into four quadrants (I–IV) and a 9-cm-diameter platform was placed in the center of quadrant I and submerged 0.5–1 cm below water surface during the initial training stage. During the initial training stage, all the mice were trained for 4 days with four trials each day. In each trial, the mouse was gently put onto water surface at a random point of each quadrant. The time spent to find the hidden platform (escape latency) of each mouse were monitored. If the mouse failed to find the platform within 60 s, the experimenter would guide the mouse and keep the mouse stay on the platform for 10 s, and the escape latency of this mouse would be recorded as 60 s. The time interval between trials was no less than 10 min to make sure all the mice got sufficient rest. After 4 days of training, all the mice were subjected to spatial probe test 24 h after the last training trial. During the spatial probe test, the platform was removed and the mouse was gently put into water at the opposite quadrant (IV). The mouse was allowed to swim freely for 60 s. The platform crossings and target quadrant dwell time (percentage of time spent in quadrant I) were recorded.

#### PD Model Mice Stimulation

The 30 PD model mice were used to further evaluate the novel stimulation method. The PD mice received a dosage of 25 mg/kg 1-methyl-4-phenyl-1,2,3,6-tetrahydropyridine (MPTP) (Patil et al., 2014) intraperitoneally (i.p.) each day for 5 days to generate PD-like behavioral phenotypes. The 30 PD mice were also divided into three groups in random: TMAS, TUS, and Con. The stimulation parameters and the experimental conditions for the PD mice were the same as those used for the healthy mice, as described in section “Healthy Mice Stimulation.” After 10 days of treatment, the 18 PD (*n* = 6 in each group) mice were subjected to *in vivo* electrophysiological experiments. And the other 12 PD mice (*n* = 4 in each group) were subjected to the MWM test to assess the spatial learning and memory abilities.

#### *In vivo* Electrophysiological Study

The mice were positioned on a stereotaxic frame (SR-6 N; Narishige, Japan) after being anesthetized with 30% (w/w) urethane (0.4 mL/kg, i.p.). A bipolar stimulating electrode and recording electrode were implanted in the perforant pathway (−3.8 mm AP, 3.0 mm ML, 1.5 mm DV) and the dentate gyrus (DG) of the hippocampus (−2.0 mm AP, 1.4 mm ML, 1.5 mm DV), respectively. A stimulation current that could evoke a field excitatory postsynaptic potential (fEPSP) of 50–60% of the maximal fEPSP was used. Long-term potentiation (LTP) was induced by theta burst stimulation (30 trains of six pulses at 100 Hz with the inter-train interval of 200 ms) after 30 min of basal fEPSP recording (once every 60 s). Then, the single stimulating pulse-evoked fEPSP was recorded every 60 s for 90 min. Afterward, depotentiation (DP) was induced by low-frequency stimulation (900 pulses of 1 Hz for 15 min), and evoked fEPSP was recorded every 60 s for 90 min (Gao et al., 2015).

## Results

### The Simulation Results Induced Electrical Field in TMAS

**FIGURE 5:**
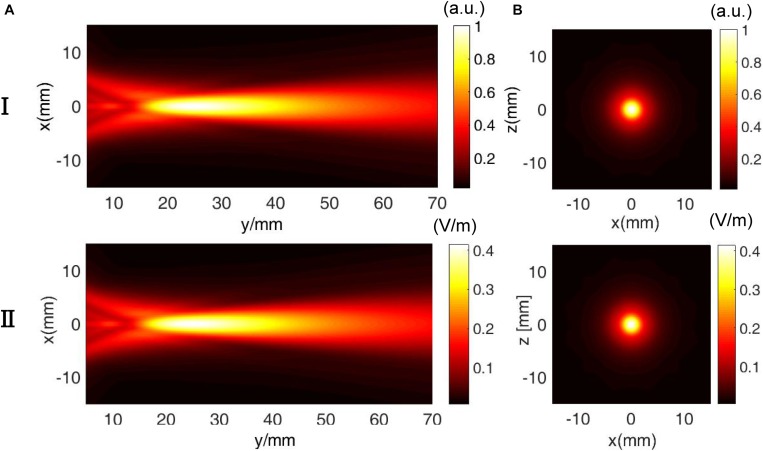
Comparison of the (I) ultrasound pressure field distribution and (II) electrical field intensity distribution at **(A)** the *x*–*y* section (*y* = 0 mm) and **(B)** *x*–*z* section (*y* = 23 mm) in the simulation.

**FIGURE 6:**
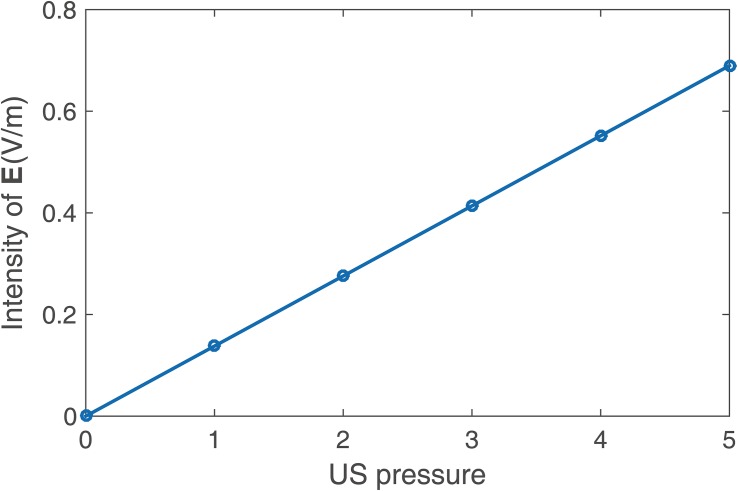
Intensity of the E-field at different US pressures in the simulation.

Figure 5 shows the (I) simulated US field distribution and (II) simulated electrical field distribution at (A) the *x*–*y* section when *y* = 0 mm and (B) the *x*–*z* section when *y* = 23 mm. The simulated focus size and focal length of the US field were nearly 2 and 23 mm, respectively (Figure 5I), and the focus size and focal length of the induced **E** (Figure 5II) were also 2 and 23 mm, respectively. The distribution of **E** was highly consistent with the distribution of the US pressure field of the transducer in a homogeneous medium. As shown in Figure 5II, the maximal electrical field intensity was 0.4 V/m when *P* = 3 MPa and *B*_0_ = 0.2 *T*. Figure 6 shows the linear relationship between the intensity of the E-field and the US pressure. Clearly, a stronger E-field was obtained as the US pressure increased.

### Measurement of the Induced Electrical Field in TMAS

**FIGURE 7:**
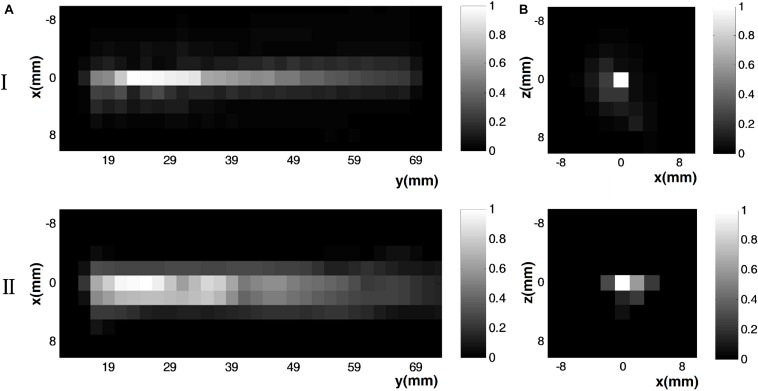
Measurement results of (I) the ultrasound pressure field distribution and (II) the electrical field intensity distribution at **(A)** the *x*–*y* section (*y* = 0 mm) and **(B)** *x*–*z* section (*y* = 23 mm) by normalization.

**FIGURE 8:**
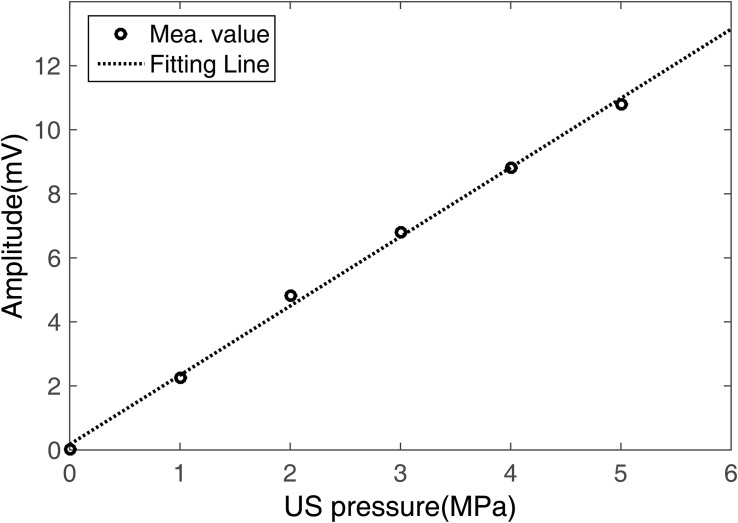
Induced voltage of the test sample at different US pressures.

Figure 7 shows measurements of (I) the ultrasound pressure field distribution and (II) the electrical field intensity distribution at (Figure 7A) the *x*–*y* section when *y* = 0 and (Figure 7B) the *x*–*z* section when *y* = 23 mm by normalization. The focus size and focal length of the US field (Figure 7I) were nearly 2 and 23 mm, respectively, as were the focus size and focal length of the induced **E** (Figure 5II). The distribution of **E** was consistent with the distribution of the US pressure field of the transducer using the test sample. Figure 8 shows the relationship between the induced voltage and the US pressure. The induced voltage (E-field) was linearly correlated with the stimulated US pressure, the same as in the simulation.

### Behavioral Results From Stimulation Experiments in Healthy Mice

**FIGURE 9:**
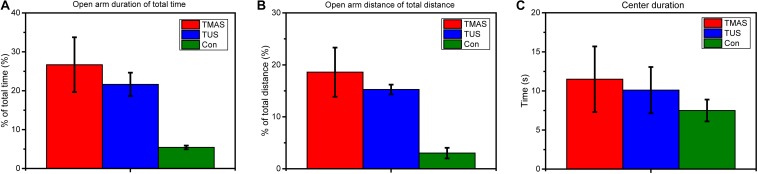
Elevated plus-maze test and open field behavior test results from stimulation experiments in healthy mice (*n* = 3). **(A)** The open arm duration (the time that mouse spent in the open arms during the test) of total time (%) and **(B)** the open arm distance (the distance that mouse traveled in the open arms during test) of total distance (%) in the elevated plus-maze test. **(C)** The center duration time (the time that mouse spent in the center area) in the open field behavior test.

Figure 9 illustrates the behavior results of the stimulation experiments on nine healthy mice described in section “Healthy Mice Stimulation.” The behavior tests included the elevated plus-maze test and the open field behavior test. Figures 9A,B show the average open arm duration as a percentage of the total time, and the average open arm distance as a percentage of the total distance in the elevated plus-maze test. Figure 9C shows the average center duration in the open field behavior test.

As shown in Figures 9A,B, both the duration (%) and the distance (%) of the TMAS group and the TUS group were higher than those of the Con group. Moreover, the duration (%) and the distance (%) of the TMAS group exceeded those of the TUS group by about 23 and 22%, respectively. As shown in Figure 9C, the center duration times of the TMAS group and TUS group were higher than those of the Con group, while the center duration of the TMAS group exceeded the TUS group by about 14%.

**FIGURE 10:**
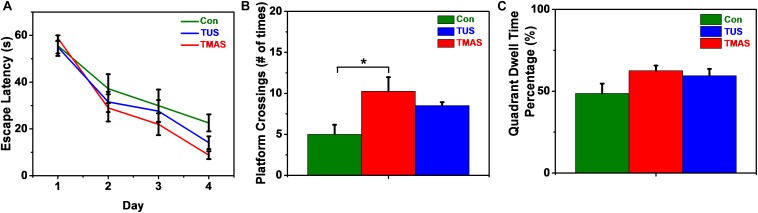
MWM training and test results of the healthy mice (*n* = 3). **(A)** Escape latencies during training days. **(B)** Platform crossings on probing test day. **(C)** Percentage of duration spent in the target quadrant. ^∗^*p* < 0.05.

As shown in Figure 10A, in the training process, the escape latencies in all groups decreased in the following days as compared to the first training day. And the rate of descent of the TMAS group is greater than the TUS group and the Con group. The rate of descent of the TUS group is greater than the Con group, too. Furthermore, the average escape latency in TMAS group is 29.64 ± 3.35 s, which is much shorter than the average escape latency in Con group (36.34 ± 5.29 s) and shorter than the average escape latency in TUS group (32.08 ± 3.60 s). In the probing test process, the platform crossings and the percentage of duration spent in the target quadrant were analyzed, as shown in Figures 10B,C. For the platform crossings results (Figure 10A), the difference between the TMAS group and Con group was significant (*p* = 0.0166). And the platform crossings times of the TMAS group (10.25 ± 1.72) is more than the TUS group (8.50 ± 0.42) and more than the Con group (5.00 ± 1.16). For the percentage of duration spent in the target quadrant results (Figure 10C), the TMAS group (62.51 ± 3.13%) is more than the TUS group (59.46 ± 4.14%) and more than the Con group (48.64 ± 5.99%). The results in Figures 10B,C show the same changing trend as those in Figure 10A.

Besides, both the healthy mice involved in behavioral tests were in good physical and mental state during the 10 days treatment and didn’t show any obvious discomfort after the TMAS and TUS.

### Electrophysiological Results and MWM Results of the Stimulation Experiments in PD Mice

**FIGURE 11:**
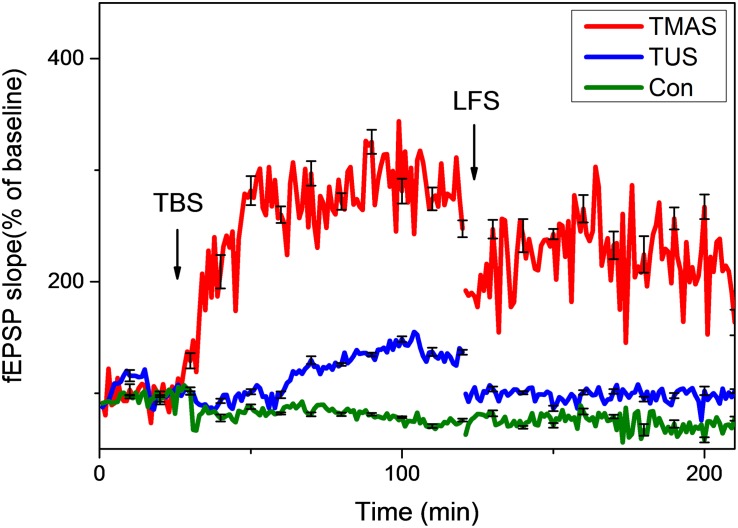
Normalized slopes of fEPSPs in hippocampus of PD mice (*n* = 6). Red line, blue line, and green line denote the TMAS group, TUS group, and Con group, respectively.

**FIGURE 12:**
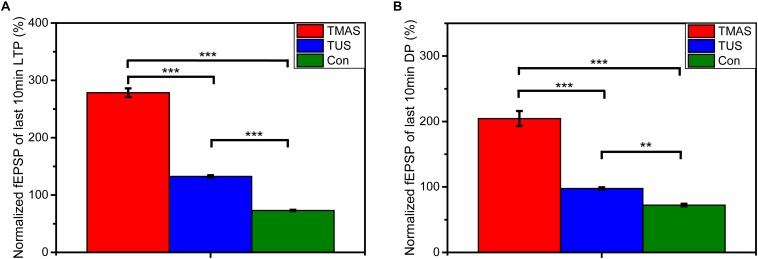
Normalized fEPSPs of **(A)** the last 10 min of LTP and **(B)** the last 10 min of DP (*n* = 6). Data were plotted as mean ± SEM. Red, blue, and green denote the TMAS group, TUS group, and Con group, respectively. ^∗∗^*p* < 0.01, ^∗∗∗^*p* < 0.001.

The results of the *in vivo* electrophysiological experiments on 18 PD mice described in section “PD Model Mice Stimulation” are shown in Figures 11, 12.

As shown in Figure 11, the TMAS treatment improved LTP substantially in PD mice. At the end of the LTP recording, the slope of fEPSP in the TMAS group reached 343.9 ± 11.6% of the baseline, which much higher than the increase seen for the Con group (74.4 ± 1.4%). The TUS treatment also improved the LTP effect in PD mice. The slope of fEPSP in the TUS group reached 154.8 ± 2.3% at the end of the LTP recording, compared with 74.4 ± 1.4% in the Con group. We quantified the extent of LTP and DP by calculating the averages of the last 10 min of LTP recording or DP recording for each group of mice, as illustrated in Figure 12. For the LTP results (Figure 12A), the difference between the TMAS group and Con group was significant (*p* < 0.001), as was the difference between the TUS group and Con group (*p* < 0.001). Moreover, there was a significant difference between the TMAS group and TUS group (*p* < 0.001). Similarly, the DP results (Figure 12B) showed significant differences between the TMAS group and Con group (*p* < 0.001), and between the TUS group and Con group (*p* < 0.01). There was significant difference between TMAS group and TUS group (*p* < 0.001), too. The results in Figure 12 show the same changing trend as those in Figure 10, and further confirm the conclusions drawn from Figure 11.

**FIGURE 13:**
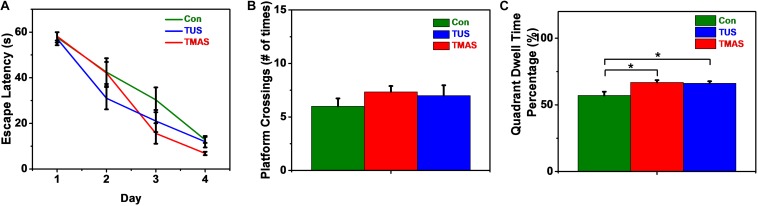
MWM training and test results of the PD mice (*n* = 4). **(A)** Escape latencies during training days. **(B)** Platform crossings on probing test day. **(C)** Percentage of duration spent in the target quadrant. ^∗^*p* < 0.05.

As shown in Figure 13A, in the training process, the escape latencies in all groups decreased in the following days as compared to the first training day. And the rate of descent of the TMAS group is greater than the TUS group and the Con group. The rate of descent of the TUS group is greater than the Con group, too. Furthermore, the average escape latency in TMAS group is 30.28 ± 3.14 s, which is much shorter than the average escape latency in Con group (35.81 ± 3.74 s) and slightly shorter than the average escape latency in TUS group (30.67 ± 3.59 s). In the probing test process, the platform crossings and the percentage of duration spent in the target quadrant were analyzed, as shown in Figures 13B,C. For the platform crossings results (Figure 13A), the platform crossings times of the TMAS group (7.33 ± 0.55) is more than the TUS group (7.00 ± 0.97) and more than the Con group (6.00 ± 0.73). For the percentage of duration spent in the target quadrant results (Figure 13C), the difference between the TMAS group and Con group was significant (*p* = 0.0143), as was the difference between the TUS group and Con group (*p* = 0.0223). And the percentage result of TMAS group (66.86 ± 1.69%) is more than the TUS group (66.17 ± 1.54%). The results in Figures 13B,C show the same changing trend as those in Figure 13A.

Besides, both the PD mice involved in electrophysiological study and MWM tests were in good physical and mental state during the 10 days treatment and didn’t show any obvious discomfort after the TMAS and TUS.

## Discussion

As shown in Figures 5 and 7, the distribution of **E** was consistent with the distribution of the US pressure field in both simulation and measurement, confirming the high-resolution ability of the TMAS method when employing a highly collimated ultrasonic beam. In our experiments, TMAS could achieve a focused electrical field with 2 mm focal size at the focal length of the transducer (23 mm). We could also acquire a series of intensities for **E** by changing the stimulated US pressure, as shown in Figures 6 and 8.

The open arm duration as a percentage of total time and the open arm distance as a percentage of total distance in the elevated plus-maze test reflect the emotion condition of mice. The center duration time in the open field behavior test reflects the active learning ability of mice. The results demonstrate the efficacy and the safety of the TMAS treatment. Limited by the sample size of the behavioral tests (three mice per treatment group), the results shown in Figure 9 only suggest that both the TMAS treatment and TUS treatment might relieve the anxiety emotion and improve the learning ability of the healthy mice. Moreover, the TMAS group showed better performance than the TUS group.

The escape latency in the MWM test reflects the spatial learning ability of mice. The platform crossings and the percentage of duration spent in the target quadrant in the MWM test reflect the spatial memory ability of mice. Similarly, limited by the sample size in the MWM tests (three mice per treatment group), the results shown in Figure 10 might suggest that both the TMAS treatment and TUS treatment might improve the learning ability and memory ability of the healthy mice. Moreover, the TMAS group showed better performance than the TUS group. The results demonstrate the efficacy and the safety of the TMAS treatment, too.

The results shown in Figures 11 and 12 illustrate that TMAS treatment and TUS treatment could potentially alleviate the memory impairments caused by MPTP treatment by improving the LTP effect in the hippocampus. The LTP effect reflects the synaptic plasticity of mice. The synaptic plasticity in hippocampus is related to the spatial learning and memory abilities. Both the TMAS treatment and TUS treatment could improve the synaptic plasticity of the PD mice. Moreover, the TMAS group showed better performance than the TUS group. The results further demonstrate the efficacy of TMAS in PD mice, as well as the safety of the TMAS treatment.

Similarly, the MWM results shown in Figure 13 suggest that both the TMAS treatment and TUS treatment might improve the spatial learning ability and memory ability of the PD mice. Moreover, the TMAS group showed better performance than the TUS group. Due to the small sample size (four mice per treatment group) in the MWM test, the MWM results could be a supplementary proof to further demonstrate the efficacy of TMAS in PD mice. The MWM results also demonstrate the efficacy and the safety of the TMAS treatment, too.

As illustrated in Figures 9–13, we could find that the synaptic plasticity of the PD mice was significantly improved by the TMAS treatments based on the electrophysiological results. And the synaptic plasticity in hippocampus is related to the spatial learning and memory abilities. According to the behavioral results, the learning and memory ability of the healthy mice might be significantly improved by the TMAS treatments. However, due to the small sample size (3 or 4 mice per treatment group) in the behavioral tests, it should admit that the behavioral results have certain limitations. So, the behavioral results could be a supplementary proof to further demonstrate the efficacy of TMAS in healthy mice and PD mice. We shall increase the sample size of the behavioral tests in the next work to improve our research. These *in vivo* results suggest that TMAS could focus on the substantia nigra of the mouse brain with a high resolution (2 mm) and have the ability to activate a small target region effectively. In addition, TUS treatments also improved the synaptic plasticity of the PD mice and might improve the learning and memory ability of the healthy mice and the PD mice compared with the Con group, although the improvement in performance of the TMAS group was superior to that of the TUS group. As described in sections “Theory of TMAS” and “Experimental Setup for TMAS,” the TMAS process inevitably contains the TUS process, based on their similar systems and the theory of TMAS. In our animal experiments, the TMAS contained a focused electrical field with *E* = 0.4 V/m and a focused US field with *I*_spta_ = 60 mW/cm^2^, simultaneously. Based on the results shown in Figures 9–13, we can speculate that the TMAS is not only a focused electrical stimulation but also a complex stimulation combining the electric field of the magneto-acoustic effect and the mechanical force of TUS, both of which play a part in TMAS treatments.

However, it is a novel finding that using an E-field with *E* = 0.4 V/m and US with *I*_spta_ = 60 mW/cm^2^ can have such a marked effect in animal experiments. When designing our *in vivo* TMAS studies, we chose the stimulation parameters (the PD, PRF, etc.) to form a proper electrical field mode by considering typical electrical stimulations. Therefore, based on recent research about TUS, the PD, the duty cycle, and the intensity of our US stimulated signal were all much lower than those of the usual stimulation wave in TUS studies (Tufail et al., 2010; Li et al., 2016; Guo et al., 2018). That is, the US stimulation used in our TMAS process was not a typical one. Furthermore, the electric field intensity (*E* = 0.4 V/m) was also much lower than the electric field intensity of typical electrical stimulations by electromagnetic induction, such as TMS and DBS. The electrical stimulation used in our TMAS experiments was really a subconvulsant electrical stimulation, since *E* = 0.4 V/m is lower than the threshold of the action potentials (Nicholls et al., 2012) of neurons.

This means that the performance of TMAS can be attributed to a combination of the electric field and the US field. The mechanism of TUS has been explored in many studies. It is generally considered that the mechanical force of US can change the threshold of ion channels in the cytomembrane (Tyler et al., 2008, 2018; Li et al., 2016; Baek et al., 2017). In the TMAS process, perpendicular mechanical forces are added to the induced E-field at the target region. The mechanical force of US waves may change the ion channels, and the E-field stimulation could achieve much better activity, accordingly. On the other hand, a perpendicular E-field is added to the mechanical force at the target region. The electrical field or electrical electricity influences the local potential or the ion channel threshold, and the US stimulation could also achieve better activity in this way. In brief, based on the *in vivo* TMAS studies, we propose that TMAS is a dual-mode stimulation, simultaneously combining an electrical stimulation by the magneto-acoustic effect and an orthogonal mechanical wave stimulation. This also provides an explanation of the mechanism of TMAS. To date, there has been no other research into the mechanism of TMAS; further studies are needed to evaluate our viewpoint.

Compared with TUS, the static magnetic field in TMAS provides an additional energy source and produces an induced E-field by the magneto-acoustic effect, which involves non-negligible neurobiological effects. TUS is an important tool in neurosciences, and is often combined with MRI and used for MRI-guided brain stimulation (Rizzitelli et al., 2015; Cho et al., 2017; Meng et al., 2017). According to the *in vivo* results, we suggest that future US stimulation in MRI-guided studies should involve careful consideration of the spatio-temporal E-field caused by the strong static magnetic field of MRI.

## Conclusion

In this paper, a novel non-invasive transcranial electrical stimulation TMAS method was, for the first time, performed experimentally and used in live mice. The induced electrical field of TMAS was simulated and measured. The results demonstrated the high-resolution ability of the TMAS method by employing a highly collimated ultrasonic beam. We built an animal experiment system, in which healthy mice and PD model mice were simulated by TMAS *in vivo*. In order to explore the relative contribution of TUS to the TMAS effect, a TUS group was added. The behavioral and electrophysiology results validate that TMAS could focus on the mouse brain with a high resolution (2 mm) and activate a small target region effectively. The results also showed that both TMAS and TUS improved the synaptic plasticity of the PD mice and might improve the learning and memory ability of the healthy mice and the PD mice, with the TMAS group being superior to the TUS group in terms of improvement in performance. Based on the *in vivo* TMAS studies, we propose that TMAS is a dual-mode stimulation combined by the electric field of the magneto-acoustic effect and the mechanical force of TUS. Our results also provide an explanation of the mechanism of TMAS. In addition, we suggest that in the future, US stimulation in MRI-guided studies should involve careful consideration of the spatio-temporal E-field caused by the strong static magnetic field.

## Ethics Statement

This study was carried out in accordance with the recommendations of the practices outlined in the National Institutes of Health Guide for the Care and Use of Laboratory Animals. The protocol was approved by the Committee for Animal Care of Nankai University.

## Author Contributions

XZ set up the experiment system, prepared the experiments, analyzed the experimental data and drafted the manuscript. SL carry out the animal stimulations and the behavioral tests. YW prepared the PD model mice and performed the electrophysiological experiments. TY designed the initial experimental system and gave many advices to this method. ZY conceived the animal experiments. ZL proposed this study and design the whole research programme. All authors revised the final version of this manuscript.

## Conflict of Interest

The authors declare that the research was conducted in the absence of any commercial or financial relationships that could be construed as a potential conflict of interest.

## References

[B1] Amiaz R. Vainiger D. Gershon A. A. Weiser M. Lavidor M. Javitt D. C. (2016). Applying transcranial magnetic stimulation (TMS) over the dorsal visual pathway induces schizophrenia-like disruption of perceptual closure. *Brain Topogr.* 29 552–560. 10.1007/s10548-016-0487-1 27021230

[B2] Aulich D. (1976). Escape versus exploratory activity: an interpretation of rats’ behavior in the open field and a light-dark preference test. *Behav. Process.* 1 153–164. 10.1016/0376-6357(76)90035-8 24923645

[B3] Baek H. Pahk K. J. Kim H. (2017). A review of low-intensity focused ultrasound for neuromodulation. *Biomed. Eng. Lett.* 7 135–142. 10.1007/s13534-016-0007-y 30603160 PMC6208465

[B4] Bergmann T. O. Karabanov A. Hartwigsen G. Thielscher A. Siebner H. R. (2016). Combining non-invasive transcranial brain stimulation with neuroimaging and electrophysiology: current approaches and future perspectives. *Neuroimage* 140 4–19. 10.1016/j.neuroimage.2016.02.012 26883069

[B5] Borckardt J. J. Smith A. R. Hutcheson K. Johnson K. Nahas Z. Anderson B. (2006). Reducing pain and unpleasantness during repetitive transcranial magnetic stimulation. *J. ECT* 22 259–264. 10.1097/01.yct.0000244248.40662.9a 17143157

[B6] Cho H. Pahk K. Kim H. (2017). Development of low-intensity focused ultrasound (LIFU) based MRI-compatible brain stimulation system. *Brain Stimul.* 10:386 10.1016/j.brs.2017.01.139

[B7] Deng Z. D. Lisanby S. H. Peterchev A. V. (2013). Electric field depth–focality tradeoff in transcranial magnetic stimulation: simulation comparison of 50 coil designs. *Brain Stimul.* 6 1–13. 10.1016/j.brs.2012.02.005 22483681 PMC3568257

[B8] Fregni F. Pascual-Leone A. (2007). Technology insight: noninvasive brain stimulation in neurology—perspectives on the therapeutic potential of rTMS and tDCS. *Nat. Rev. Neurol.* 3 383–393. 10.1038/ncpneuro0530 17611487

[B9] Gao J. Zhang X. Yu M. Ren G. Yang Z. (2015). Cognitive deficits induced by multi-walled carbon nanotubes via the autophagic pathway. *Toxicology* 337 21–29. 10.1016/j.tox.2015.08.011 26327526

[B10] Grossman N. Bono D. Dedic N. Kodandaramaiah S. B. Rudenko A. Suk H. J. (2017). Noninvasive deep brain stimulation via temporally interfering electric fields. *Cell* 169 1029.e16–1041.e16. 10.1016/j.cell.2017.05.024 28575667 PMC5520675

[B11] Guo H. Hamilton I. I. M. Offutt S. J. Gloeckner C. D. Li T. Kim Y. (2018). Ultrasound produces extensive brain activation via a cochlear pathway. *Neuron* 98 1020.e4–1030.e4. 29804919 10.1016/j.neuron.2018.04.036

[B12] Hongmiao Z. Jun L. Shaohua Y. Zhengguo L. Guang L. (2005). Measurement of bioelectric currents based on the coupling of electric and magnetic field. *Conf. Proc. IEEE Eng. Med. Biol. Soc.* 3 3001–3003. 17282874 10.1109/IEMBS.2005.1617105

[B13] Khabarova E. A. Denisova N. P. Dmitriev A. B. Slavin K. Verhagen M. L. (2018). Deep brain stimulation of the subthalamic nucleus in patients with Parkinson disease with prior pallidotomy or thalamotomy. *Brain Sci.* 8:E66. 10.3390/brainsci8040066 PMC5924402 29659494

[B14] Li G. F. Zhao H. X. Zhou H. Yan F. Wang J. Y. Xu C. X. (2016). Improved anatomical specificity of non-invasive neuro-stimulation by high frequency (5 MHz) ultrasound. *Sci. Rep.* 6:24738. 10.1038/srep24738 27093909 PMC4837374

[B15] Loo C. K. Mitchell P. B. (2005). A review of the efficacy of transcranial magnetic stimulation (TMS) treatment for depression, and current and future strategies to optimize efficacy. *J. Affect. Disord.* 88 255–267. 10.1016/j.jad.2005.08.001 16139895

[B16] Lu M. K. Chiou S. M. Ziemann U. Huang H. C. Yang Y. W. Tsai C. H. (2015). Resetting tremor by single and paired transcranial magnetic stimulation in Parkinson’s disease and essential tremor. *Clin. Neurophysiol.* 126 2330–2336. 10.1016/j.clinph.2015.02.010 25792076

[B17] Markovitz C. D. Smith B. T. Gloeckner C. D. Lim H. H. (2015). Investigating a new neuromodulation treatment for brain disorders using synchronized activation of multimodal pathways. *Sci. Rep.* 5:9462. 10.1038/srep09462 25804410 PMC4372796

[B18] Meng Y. Huang Y. Solomon B. Hynynen K. Scantlebury N. Schwartz M. L. (2017). MRI-guided focused ultrasound thalamotomy for patients with medically-refractory essential tremor. *J. Vis. Exp.* 130:e56365 10.3791/56365 29286434 PMC5755564

[B19] Nicholls J. G. Martin A. R. Wallace B. G. Fuchs P. A. (2012). *From Neuron to Brain*, 5th Edn Sunderland, MA: Sinauer Associates.

[B20] Norton S. J. (2003). Can ultrasound be used to stimulate nerve tissue? *Biomed. Eng. Online* 2:6. 12702213 10.1186/1475-925X-2-6 PMC153496

[B21] Okonogi T. Nakayama R. Sasaki T. Ikegaya Y. (2018). Characterization of peripheral activity states and cortical local field potentials of mice in an elevated plus maze test. *Front. Behav. Neurosci.* 12:62. 10.3389/fnbeh.2018.00062 29666572 PMC5891585

[B22] Patil S. P. Jain P. D. Ghumatkar P. J. Tambe R. Sathaye S. (2014). Neuroprotective effect of metformin in MPTP-induced Parkinson’s disease in mice. *Neuroscience* 277 747–754. 10.1016/j.neuroscience.2014.07.046 25108167

[B23] Pereira E. A. Green A. L. Nandi D. Aziz T. Z. (2007). Deep brain stimulation: indications and evidence. *Exp. Rev. Med. Dev.* 4 591–603. 10.1586/17434440.4.5.591 17850194

[B24] Rizzitelli S. Giustetto P. Cutrin J. C. Castelli D. D. Boffa C. Ruzza M. (2015). Sonosensitive theranostic liposomes for preclinical in vivo MRI-guided visualization of doxorubicin release stimulated by pulsed low intensity non-focused ultrasound. *J. Control Release* 202 21–30. 10.1016/j.jconrel.2015.01.028 25626083

[B25] Rotenberg A. Horvath J. C. Pascual-Leone A. (2014). *Transcranial Magnetic Stimulation.* New York: Springer.

[B26] Sato T. Shapiro M. G. Tsao D. Y. (2018). Ultrasonic neuromodulation causes widespread cortical activation via an indirect auditory mechanism. *Neuron* 98 1031.e5–1041.e5. 10.1016/j.neuron.2018.05.009 29804920 PMC8127805

[B27] Theodore W. H. Hunter K. Chen R. Vega–Bermudez F. Boroojerdi B. Reeves–Tyer P. (2002). Transcranial magnetic stimulation for the treatment of seizures: a controlled study. *Neurology* 59 560–562. 12196649 10.1212/wnl.59.4.560

[B28] Tufail Y. Matyushov A. Baldwin N. Tauchmann M. L. Georges J. Yoshihiro A. (2010). Transcranial pulsed ultrasound stimulates intact brain circuits. *Neuron* 66 681–694. 10.1016/j.neuron.2010.05.008 20547127

[B29] Tufail Y. Yoshihiro A. Pati S. Li M. M. Tyler W. J. (2011). Ultrasonic neuromodulation by brain stimulation with transcranial ultrasound. *Nat. Protoc.* 6 1453–1470. 10.1038/nprot.2011.371 21886108

[B30] Tyler W. J. Shane W. L. Grace M. H. (2018). Ultrasonic modulation of neural circuit activity. *Curr. Opin. Neurobiol.* 50 222–231. 10.1016/j.conb.2018.04.011 29674264

[B31] Tyler W. J. Tufail Y. Finsterwald M. Tauchmann M. L. Olson E. J. Majestic C. (2008). Remote excitation of neuronal circuits using low-intensity, low-frequency ultrasound. *PLoS One* 3:e3511. 10.1371/journal.pone.0003511 18958151 PMC2568804

[B32] Vorhees C. V. Williams M. T. (2006). Morris water maze: procedures for assessing spatial and related forms of learning and memory. *Nat. Protoc.* 1 848–858. 10.1038/nprot.2006.116 17406317 PMC2895266

[B33] Yuan Y. Chen Y. Li X. (2016). Theoretical analysis of transcranial magneto-acoustical stimulation with Hodgkin-Huxley neuron model. *Front. Comput. Neurosci.* 10:35. 10.3389/fncom.2016.00035 27148032 PMC4835452

[B34] Zhang S. Cui K. Zhang X. Shi X. Ge M. Zhao M. (2018). Effect of transcranial ultrasonic–magnetic stimulation on two types of neural firing behaviors in modified Izhikevich model. *IEEE Trans. Magnet.* 54 1–4. 10.1109/tmag.2017.2773086

